# Coronary stent fixation of left ventricular lead during CRT-D implantation: a case series of three patients

**DOI:** 10.3389/fcvm.2026.1984315

**Published:** 2026-10-14

**Authors:** Hongwei Yi, Jia Li, Yue Bao, Hongwei Han

**Affiliations:** Department of Cardiology, Wuhan Asian Heart Hospital, Wuhan, Hubei, China

**Keywords:** cardiac resynchronization therapy defibrillator, coronary sinus, coronary stent, heart failure, lead dislodgement, left ventricular lead, off-label use

## Abstract

**Objective:**

To investigate a salvage method using coronary stent fixation of the left ventricular (LV) lead to manage refractory LV lead dislodgement caused by unfavorable coronary venous anatomy during cardiac resynchronization therapy defibrillator (CRT-D) implantation, and to evaluate its technical feasibility and clinical value.

**Methods:**

We retrospectively included three patients with heart failure with reduced ejection fraction (HFrEF) at our center who developed LV lead dislodgement during or early after CRT-D implantation. Conventional methods failed to maintain a stable lead position. A sirolimus-eluting coronary stent was deployed within the target coronary vein to achieve mechanical anchoring of the LV lead via gentle compression against the venous wall. Procedural details, perioperative complications, and follow-up data were collected and analyzed.

**Results:**

All three patients completed stent fixation of the LV lead. No acute severe complications, such as coronary venous rupture, pericardial tamponade, or lead conductor damage, occurred intraoperatively. No significant change in pacing threshold was observed before and after stent deployment. Cases 1 and 2 were followed for 30 and 26 months, respectively, and lead parameters remained stable. Case 3 was followed for only 2 months and then lost to follow-up; lead parameters were stable at that time point. No clinically meaningful improvement in LV ejection fraction (LVEF) was observed in Cases 1 and 2 during follow-up. Because Case 3 was followed for only 2 months, shorter than the usual 6–12-month window for assessing CRT reverse remodeling, functional response could not be evaluated. This procedure constitutes off-label device use; after full stent deployment, the lead position cannot be readjusted. If device-related infection occurs, transvenous lead extraction may be difficult and uncertain, and surgical removal may be required.

**Conclusions:**

After all conventional methods have failed, stent fixation may serve as a salvage option for refractory LV lead dislodgement caused by unfavorable coronary venous anatomy. This report describes the clinical observations of sirolimus-eluting stent use in this off-label salvage procedure and details the relevant procedural parameters and techniques. However, the sample size is small, and potential long-term risks, including stent fracture, lead insulation damage, and stability of lead electrical parameters, require further evaluation in larger studies. This technique only addresses the mechanical fixation of the lead and does not guarantee functional benefit from CRT.

## Introduction

After more than 20 years of development, cardiac resynchronization therapy (CRT) has become a mature treatment for heart failure. Although conduction system pacing (CSP), particularly His bundle pacing and left bundle branch pacing, offers certain advantages in improving cardiac function (1), some patients still cannot achieve ideal pacing outcomes because of operator experience and anatomical limitations. Therefore, biventricular pacing remains of irreplaceable clinical value.

When the coronary venous branch anatomy is unfavorable, especially when the target vein is large, straight, and has a wide angle with the coronary sinus, reliable mechanical anchoring of a conventional passive fixation lead is difficult. Biffi et al. (2) classified coronary veins into three types according to their angle with the coronary sinus: Type A veins originate between the coronary sinus ostium and the proximal third of the coronary sinus and course upward to a lateral or posterior-lateral pacing site; Type B veins have a flat take-off from the coronary sinus at an angle >80°; Type C veins have a ≤ 80° take-off or a gooseneck take-off from the coronary sinus. Type A and B veins are high-risk anatomical types for lead dislodgement because of insufficient side-branch anchoring.

Several management strategies have been reported. For narrowed coronary veins, balloon angioplasty can be performed (3). For large target veins, a retained guidewire inside the lead can provide additional support (4), but long-term retained guidewires carry risks of fracture and wear of the lead insulation. Active fixation LV leads provide reliable fixation (5), but not all centers routinely have them available. If all interventional approaches fail, surgical epicardial lead implantation can be considered (6); however, this approach is invasive and carries high surgical risk in patients with advanced heart failure.

For salvage management of LV lead dislodgement, coronary stent fixation in the coronary venous system was first reported by Cesario et al. (7) in 2006. In 2011, Geller et al. (8) reported a large series of 312 patients with long-term experience, confirming that this technique effectively reduced the dislodgement rate (0.6%) and maintained stable mid- and long-term pacing parameters. Kamalzadeh et al. (9) also reported two clinical cases in 2018. However, all previous studies used bare-metal stents (BMS). Reports on drug-eluting stents (DES) in the coronary venous system are limited, and practical details such as stent sizing, deployment pressure, and positioning techniques are poorly documented.

At our center, three patients in whom stable anchoring of the LV lead could not be achieved by conventional methods were treated with a sirolimus-eluting coronary stent to fix the LV lead as a salvage approach, aiming to avoid the high trauma risk of surgery. Postoperative follow-up showed stable lead electrical parameters (Table 1). We report the procedural course, follow-up results, technical details, and associated risks, and compare our findings with previous studies.

**Table 1:** Baseline, procedural, and follow-up data of the three patients.

Variable	Case 1	Case 2	Case 3
Age (years)	50	63	72
Underlying cardiac disease	Dilated cardiomyopathy, hypertension	Dilated cardiomyopathy, hypertension, paroxysmal atrial fibrillation	Third-degree atrioventricular block, dilated cardiomyopathy
Preoperative LVEF (%)	30	30	35
QRS duration (ms)	146	128	254
Clinical setting of dislodgement	Lead dislodgement on postoperative day 1; second procedure for repositioning	Repeated dislodgement into the right atrium during sheath slitting	Immediate dislodgement into the coronary sinus during sheath slitting
Stent type	Sirolimus-eluting coronary stent	Sirolimus-eluting coronary stent	Sirolimus-eluting coronary stent
Stent size (mm)	2.75 × 13	3.0 × 13	3.0 × 13
Stent deployment pressure (atm)	8	8	8
Immediate postoperative LV threshold (V@0.4 ms)	0.5	1.3	1.5
Last follow-up duration	30 months	26 months	2 months (subsequently lost to follow-up)
Last follow-up LV threshold (V@0.4 ms)	1.0	0.75	1.5
Last follow-up LVEF (%)	24	33	33

Coronary stent placement in the coronary venous system is an individualized clinical salvage therapy, not a pre-designed research intervention. According to institutional policy, a single salvage off-label procedure does not require separate prospective approval by the ethics committee; however, this retrospective case series was approved by the ethics committee. All patients were fully informed before the procedure of the off-label nature, risks, and the alternative of surgical epicardial lead placement, and signed specific written informed consent.

## Case reports

### Case 1

A 50-year-old man was admitted with “chest tightness and shortness of breath for 4 years, aggravated for 1 week.” He had a history of hypertension. Physical examination revealed fine crackles in both lungs and mild bilateral lower limb edema. Electrocardiography (ECG) showed sinus rhythm, complete left bundle branch block, and a QRS duration of 146 ms. Echocardiography showed a left ventricular end-diastolic dimension (LVEDD) of 6.7 cm and LVEF of 30%. Coronary angiography showed no coronary stenosis. N-terminal pro-B-type natriuretic peptide (NT-proBNP) was 3850.10 pg/mL. The diagnosis was dilated cardiomyopathy, New York Heart Association (NYHA) functional class III. The patient had received guideline-directed optimized medical therapy for heart failure for 4 years with unsatisfactory symptom improvement and met a Class IIa indication for CRT-D implantation (10). CRT-D implantation was performed under general anesthesia on hospital day 4.

Intraoperatively, His-Purkinje system pacing was attempted but failed, and LV lead implantation via the coronary sinus was performed. Coronary sinus venography (Figure 1, Supplementary Video 1) suggested that the posterior vein of the left ventricle (Figure 2) was more suitable as the target vessel than other LV veins. A 4298 LV lead (Medtronic, Minneapolis, MN, USA) was positioned with satisfactory electrical parameters. After the delivery sheath was slit under fluoroscopic guidance, lead position appeared acceptable on live fluoroscopy. After the remaining leads were implanted and parameters were rechecked and found to be good, the CRT-D pulse generator was connected, and the pocket was closed. Postoperative ECG monitoring revealed abnormal LV pacing. Bedside chest x-ray confirmed lead dislodgement (Figure 3). A repositioning procedure was performed on postoperative day 2, and intraoperative fluoroscopy again confirmed dislodgement of the lead into the pulmonary artery (Figure 4).

**Figure 1:**
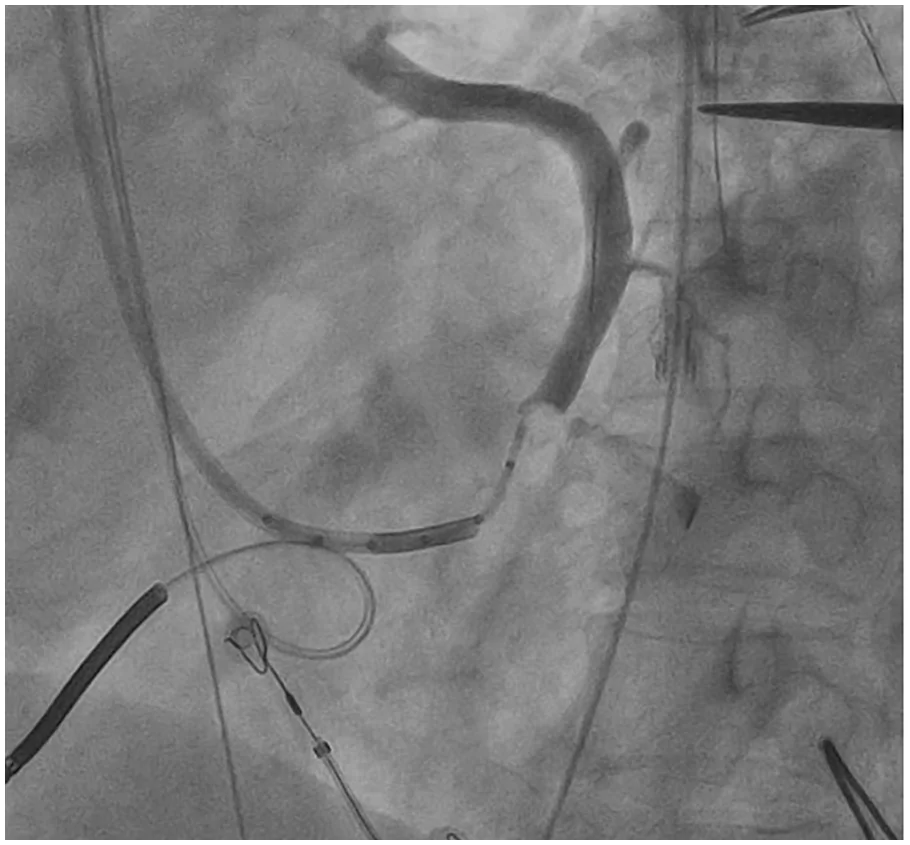
Coronary sinus and its branch veins (left posterior vein, left marginal vein, left anterolateral vein).

**Figure 2:**
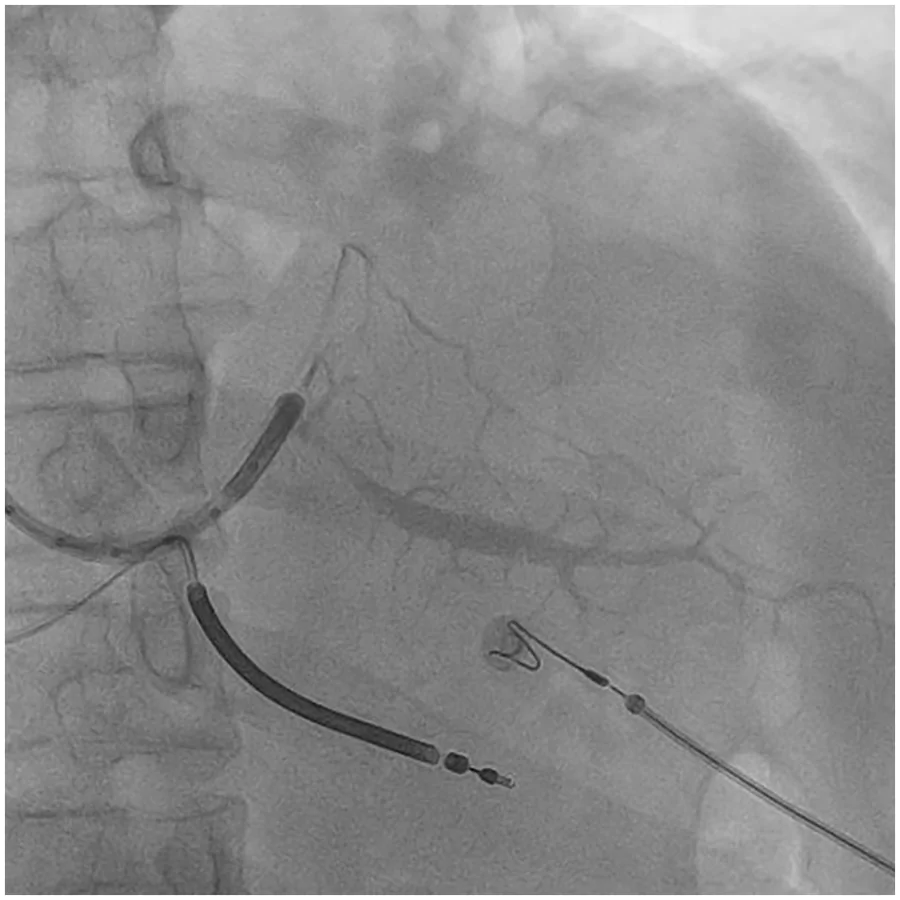
The posterior vein is large and ectatic.

**Figure 3:**
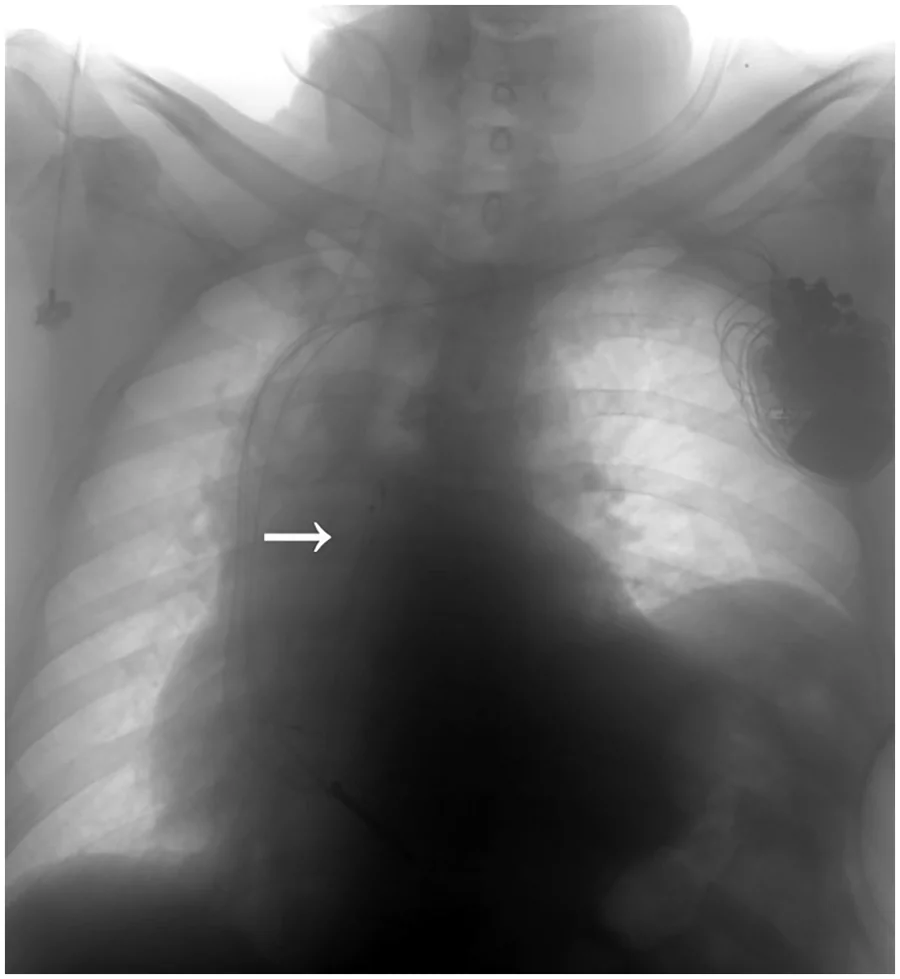
Bedside chest radiograph demonstrating lead dislodgement (acquired in reverse-contrast view).

**Figure 4:**
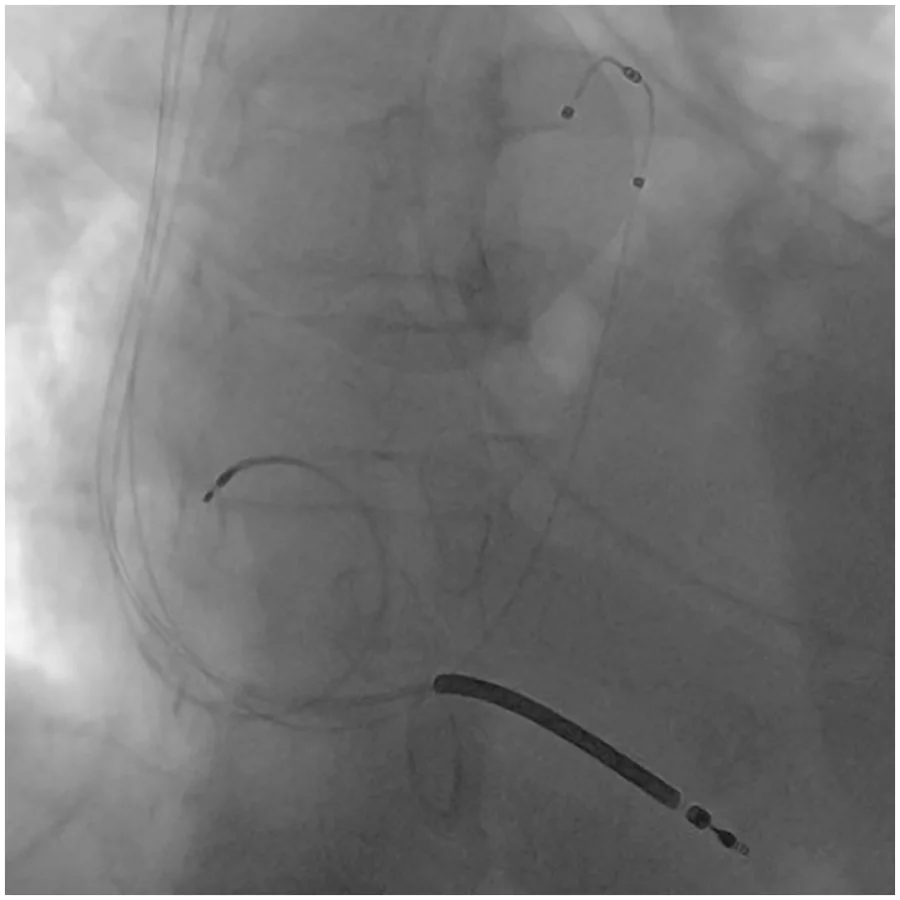
Intraoperative fluoroscopy confirming lead dislodgement into the pulmonary artery.

To prevent repeated dislodgement, the family was fully informed before the procedure that this was off-label device use and of the associated risks, and written informed consent was obtained. Coronary stent fixation of the LV lead was then performed. The initial implantation procedure was repeated, and the lead was repositioned in the posterior vein of the left ventricle. After satisfactory electrical parameters were confirmed, a Firebird sirolimus-eluting coronary stent (2.75 mm × 13 mm; MicroPort, Shanghai, China) was advanced between the third and fourth electrode rings of the lead, approximately 5 mm from the third electrode ring. Repeat measurements showed no significant change in lead parameters. The balloon was inflated at 8 atm for 5–8 s, and the stent compressed the lead against the venous wall (Figures 5 and 6, Supplementary Video 2). The delivery sheath was slit under fluoroscopy, and no lead displacement was observed (Figure 7). The repeat LV pacing threshold was 0.5 V@0.4 ms. The CRT-D pulse generator was connected, the pocket was closed, and the procedure was completed.

**Figure 5:**
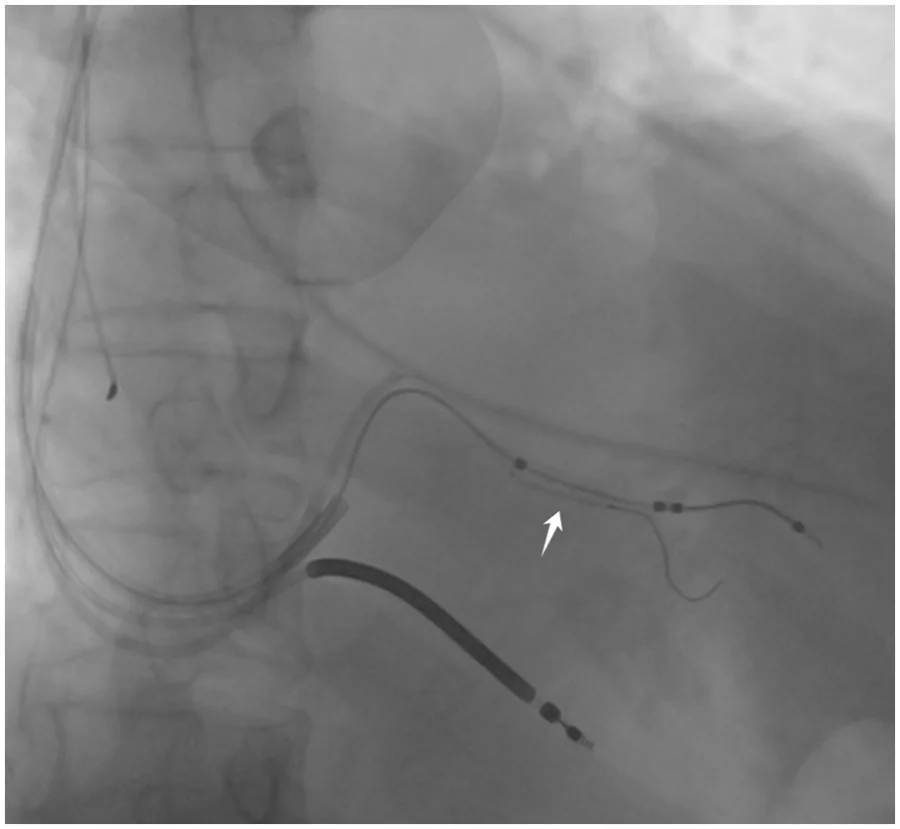
Positioning the coronary stent between the third and fourth electrode rings of the lead.

**Figure 6:**
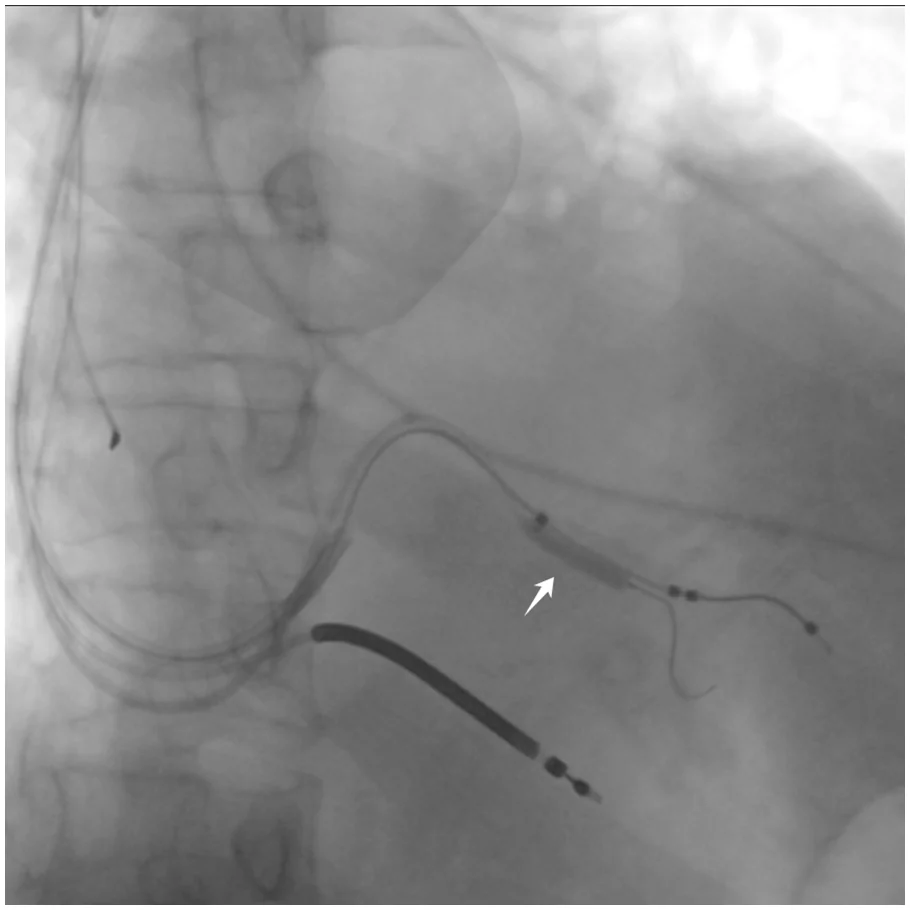
Deployment of the coronary stent.

**Figure 7:**
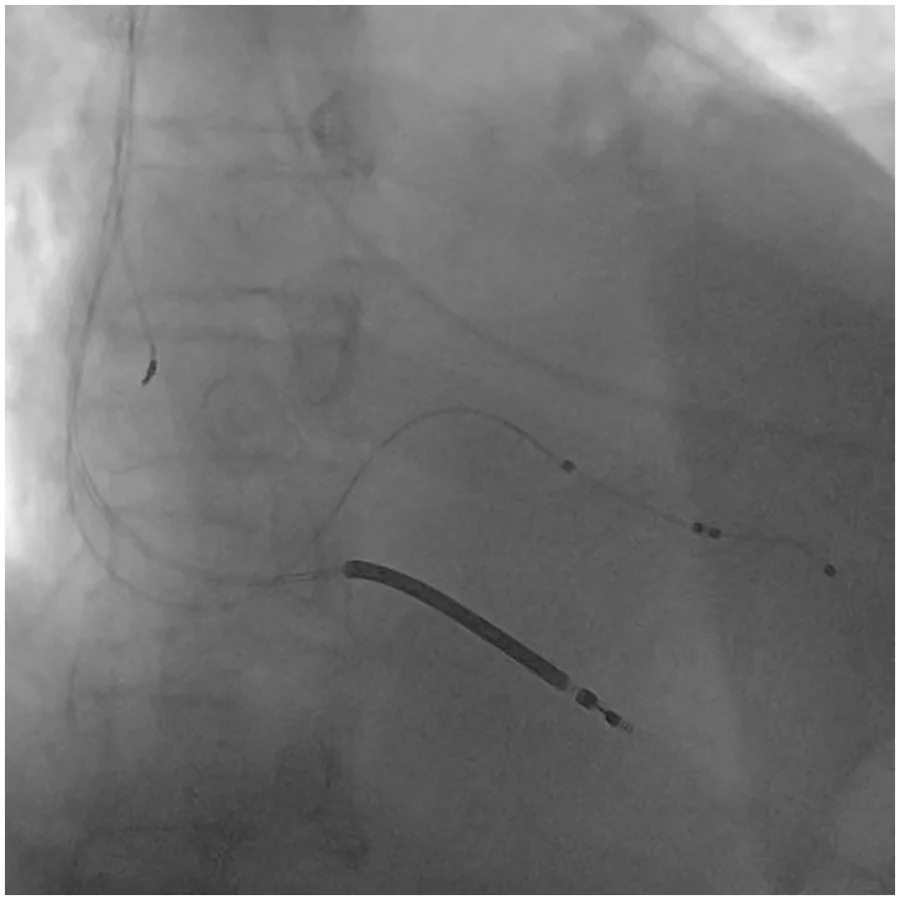
No lead dislodgement after sheath slitting.

At 30 months’ follow-up, device interrogation showed an LV lead threshold of 1.0 V@0.4 ms. Echocardiography showed LVEDD 8.0 cm and LVEF 24%.

### Case 2

A 63-year-old man was admitted with “palpitations for 1 week.” He had a 20-year history of hypertension and a 4-year history of paroxysmal atrial fibrillation. Physical examination revealed fine crackles in both lungs and an irregularly irregular rhythm. ECG showed atrial fibrillation, intraventricular conduction delay, and a QRS duration of 128 ms. Echocardiography showed LVEDD 6.7 cm and LVEF 30%. He met the indication for CRT-D implantation.

Intraoperative His-Purkinje system pacing failed. After initial coronary venography (Supplementary Video 3), a 4196 LV lead (Medtronic, Minneapolis, MN, USA) was implanted via the coronary sinus. The lead position and parameters were satisfactory; however, during slitting of the delivery sheath, the lead repeatedly dislodged into the right atrium, and stable anchoring could not be achieved despite multiple attempts (Supplementary Videos 4–6). The patient's family refused surgical epicardial lead implantation. After fully understanding the risks of off-label stent fixation of the lead, they provided informed consent.

After the LV lead was positioned and satisfactory electrical parameters were obtained, a Firebird sirolimus-eluting coronary stent (3.0 mm × 13 mm) was advanced and placed adjacent to an electrode ring, approximately 5 mm from the ring. Repeat lead parameters were unchanged. The stent was inflated at 8 atm for 5–8 s to compress and fix the LV lead. After sheath removal, the lead position was stable, and the remaining leads were implanted (Supplementary Video 7). The intraoperative LV pacing threshold was 1.3 V@0.4 ms.

At 26 months’ follow-up, device interrogation showed an LV pacing threshold of 0.75 V@0.4 ms. Echocardiography showed LVEDD 6.6 cm and LVEF 33%.

### Case 3

A 72-year-old man was admitted with “recurrent shortness of breath for 2 years, aggravated for 10 days.” He had previously received a single-chamber pacemaker for third-degree atrioventricular block. Physical examination revealed fine crackles in both lower lung fields and a blowing systolic murmur at the apex. ECG showed sinus rhythm with a ventricular paced rhythm and a QRS duration of 254 ms. Echocardiography showed LVEDD 6.3 cm and LVEF 35%. He met the indication for CRT-D upgrade.

The procedure was performed under general anesthesia. His-Purkinje system pacing was attempted but failed. An LV lead was implanted via the coronary sinus, and electrical parameters were acceptable; however, at the moment of sheath slitting, the lead dislodged into the coronary sinus, and dislodgement still occurred despite repeated adjustments. The family refused surgical epicardial lead implantation, requested an attempt at coronary stent fixation of the lead, and provided written informed consent.

The lead was again implanted in the target vein. A Firebird sirolimus-eluting coronary stent (3.0 mm × 13 mm) was advanced adjacent to an electrode ring of the lead. The stent was deployed at 8 atm to fix the lead. After sheath removal, the lead position was stable. The intraoperative LV pacing threshold was 1.5 V@0.4 ms.

At 2 months’ outpatient follow-up, the LV pacing threshold was 1.5 V@0.4 ms. Echocardiography showed LVEDD 6.5 cm and LVEF 33%. The patient was subsequently lost to follow-up, and further clinical and device interrogation data could not be collected.

### Key procedural points

#### Preoperative informed consent

Use of a coronary stent in the coronary venous system is off-label. This technique is a salvage procedure. Before the procedure, the patient and family must be fully informed of the following risks, and written informed consent must be obtained: (1) coronary vein dissection, rupture, and pericardial tamponade; (2) stent compression injury to the lead insulation and conductor coil; (3) inability to reposition the lead after full stent deployment; and (4) if device-related infection occurs, transvenous lead extraction may be difficult and uncertain, and surgical removal may be required.

#### Procedural steps

1. A 6250 VIC delivery sheath (Medtronic, Minneapolis, MN, USA) was advanced into the coronary sinus over a Lee's coronary sinus venogram balloon catheter (China). The balloon catheter was removed, and two long guidewires were advanced through the delivery sheath to the distal coronary sinus. The delivery sheath was then removed to establish a double-guidewire rail.
2. The delivery sheath was re-advanced into the coronary sinus over one of the guidewires. Under delivery sheath support, the LV lead was advanced to the target vessel using the standard technique. Pacing threshold and impedance were tested to confirm satisfactory electrical performance, and phrenic nerve stimulation was excluded.
3. A guiding catheter was advanced into the coronary sinus over the other guidewire. A 0.014-inch guidewire was advanced through the guiding catheter to the distal target vein. Over the guidewire, a balloon dilatation catheter preloaded with a coronary stent was advanced to the insulated segment of the lead in close proximity to the electrode-ring.
4. Lead electrical parameters were rechecked. If parameters were unsatisfactory, the lead position was readjusted, and the stent position was adjusted accordingly so that the stent was 5–10 mm from the adjacent electrode ring. The stent must never contact two metal electrode rings simultaneously.
5. The stent was deployed at 8 atm to compress the lead against the venous wall. Excessive pressure was avoided to prevent coronary vein rupture.
6. The balloon and guidewire were withdrawn under fluoroscopy, and the delivery sheath was gently slit and removed while observing for lead displacement in real time.
7. After all steps were completed, threshold, sensing, and impedance parameters were rechecked. The lead was sutured and fixed to the subcutaneous tissue of the pocket.

#### Stent selection principles

1. **Stent diameter:** The stent diameter was selected to be 0.25–0.5 mm smaller than the measured target vein diameter, aiming to effectively anchor the lead without causing excessive venous dilatation injury.
2. **Stent length:** A short stent of 8–13 mm is preferred. It should cover the insulated segment of the lead, in the immediate vicinity of the electrode-ring with satisfactory pacing parameters, so that the electrode-ring can appose closely against the venous vessel wall.

#### Tips and pitfalls

1. **Avoid electrode rings:** The stent must never contact two electrode rings simultaneously to avoid short-circuiting between the electrode rings. Multi-angle fluoroscopy must be used to confirm the position before deployment. The stent should be kept 5–10 mm from the electrode ring. If the distance is too great, the adjacent electrode ring may lift-off from the venous wall, leading to increased pacing threshold.
2. **Stent size:** If the stent diameter is too small, anchoring strength is insufficient. If the diameter is too large, the coronary vein may be injured. If the stent is too short, anchoring may be unstable, or the electrode rings on both sides of the stent may lift-off, resulting in poor apposition to the vessel wall and increased threshold. If the stent is too long, it increases the risk of venous injury and future extraction difficulty.
3. **Sheath slitting:** After stent deployment, withdraw the balloon and guidewire first, then slit the sheath under fluoroscopy. Before slitting, reserve sufficient lead length in the coronary sinus to avoid sheath traction causing lead displacement. Alternatively, slit the sheath first and then withdraw the balloon and guidewire. This allows additional higher-pressure dilation if inadequate stent expansion or an undersized stent causes unstable lead fixation.

## Discussion

For patients with heart failure with reduced ejection fraction whose symptoms remain difficult to control despite optimal medical therapy, CRT is an important guideline-recommended treatment that improves ventricular synchrony, relieves heart failure symptoms, and reduces heart failure rehospitalization (10). Although His-Purkinje system pacing has advantages in improving cardiac function, some patients cannot achieve correction of left bundle branch block or do not meet left bundle branch pacing criteria; therefore, biventricular pacing remains of important clinical value. Difficult lead fixation caused by unfavorable target coronary venous anatomy remains a technical challenge during LV lead implantation.

For LV lead implantation difficulties caused by coronary venous anatomical abnormalities, several interventional techniques have been developed, including active fixation leads such as the 4798 (Medtronic, Minneapolis, MN, USA), snare technique (11), anchor balloon technique (12), Jurdham procedure (13), coronary venous angioplasty, retained guidewire technique, and use of leads with different curves to accommodate different veins. However, each method has its own limitations. When all interventional approaches fail, surgical epicardial lead implantation is the only remaining option.

Coronary stent fixation of the LV lead as a salvage treatment for LV lead dislodgement has been reported in a series of studies. Previous large studies were mostly based on bare-metal stents and confirmed that this method significantly reduces the incidence of lead dislodgement (7–9). In managing these three high-risk cases, our center drew on previous literature experience. Because bare-metal stents were unavailable, we used a sirolimus-eluting stent and made corresponding modifications based on clinical practice.

All three patients underwent standard transvenous LV lead implantation via the coronary sinus, but lead dislodgement occurred intraoperatively or early postoperatively. The main reasons were a large, straight target vein with insufficient anchoring points and a wide angle between the delivery sheath and the lead during sheath slitting, which caused friction and traction that predisposed to lead displacement. Active fixation LV leads theoretically avoid such dislodgement, but such devices were not available at our center at that time. When no other feasible alternative existed, stent fixation was chosen as a salvage approach to prevent repeated dislodgement. We selected a sirolimus-eluting stent (DES). No additional antiplatelet or anticoagulant regimen was added postoperatively; instead, the patient's pre-existing baseline anticoagulant or antithrombotic strategy was continued, consistent with the management principles of the large series by Geller et al. (8). Lead electrical parameters remained stable during follow-up, preliminarily suggesting short-term safety of DES use in the coronary venous system.

However, echocardiography during follow-up showed no clinically meaningful improvement in LVEF in any of the three patients. Possible reasons include the following: all three patients had end-stage dilated cardiomyopathy with extensive myocardial fibrosis, and the probability of CRT reverse remodeling was inherently low; Case 2 did not have typical complete left bundle branch block, and CRT response was limited; and concomitant valvular regurgitation and other underlying cardiac pathologies further limited functional improvement. Case 3 was lost to follow-up after only 2 months, which is shorter than the 6–12-month evaluation window for CRT reverse remodeling, so CRT response could not be assessed. These results suggest that stent fixation of the lead only solves the mechanical fixation problem of the lead; even if stable electrical parameters are obtained, it cannot guarantee functional benefit from CRT. Preoperative expectations should be objectively communicated to patients and families.

Compared with previous large case series using bare-metal stents, this case series provides clinical observational data on a sirolimus-eluting stent used in this off-label salvage procedure. It also details a reproducible set of procedural parameters, including stent–lead relative position, stent size matching principles, balloon inflation pressure and duration, and sheath-slitting techniques, which may provide a reference for clinical practice. Two patients had mid- to long-term follow-up of 26–30 months, adding real-world observational data regarding lead electrical stability with this specific stent-lead combination. This technique can serve as a minimally invasive method to manage lead dislodgement and avoid the high risk and trauma of surgical thoracotomy; however, it is only suitable as a salvage option after other approaches have been exhausted and is not recommended as a first-line clinical choice. For the few cases with unfavorable target vessel anatomy, high predicted risk of dislodgement, and no availability of active fixation leads, this salvage approach may be considered. However, the number of cases in this series is limited, and the safety, efficacy, and long-term stability of the lead still require longer observation.

### Limitations

This study is a single-center retrospective case series with a small sample size and no control group. Coronary stent placement in the coronary venous system is off-label, and endpoints such as long-term stent fracture, lead insulation damage, and stability of lead electrical parameters could not be evaluated. CRT non-response may be due to end-stage organic myocardial injury rather than an implantation technique issue. One patient was lost to follow-up after only short-term follow-up, so CRT functional response could not be evaluated, further lowering the level of evidence for mid- and long-term safety. Future multicenter studies with larger numbers of cases are needed to further define the indications, contraindications, and long-term safety of this technique. In addition, bioabsorbable stents could theoretically enable transvenous lead extraction after providing temporary anchoring before degradation. Nevertheless, no clinical data support this concept; therefore, this approach cannot be recommended for clinical use.

## Data Availability

The raw data supporting the conclusions of this article will be made available by the authors, without undue reservation.

## Supplementary material

SUPPLEMENTARY VIDEO 1 Left anterior oblique and right anterior oblique delayed coronary venography showing that the posterior vein is large and ectatic.

SUPPLEMENTARY VIDEO 2 Stent dilation for left ventricular lead fixation.

SUPPLEMENTARY VIDEO 3 Coronary venography showing that the posterior vein.

SUPPLEMENTARY VIDEO 4 First attempt at lead fixation and subsequent lead dislodgement.

SUPPLEMENTARY VIDEO 5 Second attempt at lead fixation and subsequent lead dislodgement.

SUPPLEMENTARY VIDEO 6 Third attempt at lead fixation and subsequent lead dislodgement.

SUPPLEMENTARY VIDEO 7 No further lead dislodgement after stent fixation of the lead.
